# Fresh, dried or smoked? repellent properties of volatiles emitted from ethnomedicinal plant leaves against malaria and yellow fever vectors in Ethiopia

**DOI:** 10.1186/1475-2875-10-375

**Published:** 2011-12-19

**Authors:** Fitsum Fikru Dube, Kassahun Tadesse, Göran Birgersson, Emiru Seyoum, Habte Tekie, Rickard Ignell, Sharon R Hill

**Affiliations:** 1 Division of Chemical Ecology, Department of Plant Protection Biology, Swedish University of Agricultural Sciences, 230 53 Alnarp, Sweden; 2 Department of Plant Science, McGill University, Lakeshore Road, Ste-Anne-de-Bellevue, Québec H9X 3V9, Canada; 3 Department of Biology, Addis Ababa University, PO Box 1176, Addis Ababa, Ethiopia

## Abstract

**Background:**

In the search for plant-based mosquito repellents, volatile emanations were investigated from five plant species, *Corymbia citriodora*, *Ocimum suave*, *Ocimum lamiifolium, Olea europaea *and *Ostostegia integrifolia*, traditionally used in Ethiopia as protection against mosquitoes.

**Methods:**

The behaviour of two mosquitoes, the malaria vector *Anopheles arabiensis *and the arbovirus vector *Aedes aegypti*, was assessed towards volatiles collected from the headspace of fresh and dried leaves, and the smoke from burning the dried leaves in a two-choice landing bioassay and in the background of human odour.

**Results:**

Volatile extracts from the smoke of burning dried leaves were found to be more repellent than those from fresh leaves, which in turn were more repellent to mosquitoes than volatiles from dried leaves. Of all smoke and fresh volatile extracts, those from *Co. citriodora *(52-76%) and *Oc. suave *(58-68%) were found to be the most repellent, *Os. integrifolia *(29-56%) to be intermediate while *Ol. europaea *(23-40%) and *Os. integrifolia *(19-37%) were the least repellent. One volatile present in each of the fresh leaf extracts of *Co. citriodora*, *Oc. suave *and *Os. integrifolia *was ß-ocimene. The levels of ß-ocimene reflected the mosquito repellent activity of these three fresh leaf extracts. Female host-seeking mosquitoes responded dose-dependently to ß-ocimene, both physiologically and behaviourally, with a maximal behavioural repulsion at 14% ß-ocimene. ß-ocimene (14%) repels mosquitoes in our 6-minute landing assays comparable to the synthetic insect repellent N,N-diethyl-m-toluamide (10% DEET).

**Conclusions:**

Volatiles in the smoke of burning as well as fresh leaves of *Co. citriodora *and *Oc. suave *have significant repellent properties against host seeking *An. arabiensis *and *Ae. aegypti *mosquitoes. ß-ocimene, present in the fresh leaf headspace of *Co. citriodora*, *Oc. suave *and *Os. integrifolia*, is a significantly effective volatile mosquito repellent in the laboratory. In addition to its repellent properties, ß-ocimene has long approved safe for use in food and cosmetics, making this volatile an intriguing compound to pursue in further tests in the laboratory and field to validate its mosquito repellent activity and potential for use in a commercial product. Also, the landing bioassay with humanised membranes is a potentially useful repellent screening technique that does not require the exposure of humans to the vectors, however further tests in parallel with conventional techniques are advised.

## Background

The health risks associated with arthropod disease vectors have long encouraged research into methods for protection in endemic areas, in both the grassroots [1] and scientific communities. Diligent investigations into such grassroots protection methods by the scientific community is leading to the development of new bio-rational, effective and affordable products as well as increasing knowledge and confidence in traditional protection methods and reducing vector-borne disease.

One of the most effective strategies to minimize vector-borne disease is personal protection, which focuses on the behaviour of both people and mosquitoes to minimize human exposure to vectors [2]. The use of insecticide-treated nets (ITNs) is the most powerful method for personal protection currently available for effective infection reduction [3]. Even so, ITNs have their limitations; primarily, that they do not protect against exophagic vectors, or those vectors that bite at times when people are not sleeping under their bed nets. [4].

Disease vectors, such as *Anopheles arabiensis*, the primary malaria vector in semi-arid eastern sub-Saharan Africa [5,6], *Aedes aegypti*, the main vector of dengue and yellow fever, as well as the malaria vectors *Anopheles farauti sensu lato *and *Anopheles darlingi *[7,8], have adapted their peak biting activities to the early evening and early morning, when their potential hosts are less protected. In fact in some regions, *An. darlingi *has become exclusively exophagic, arguably in response to indoor residual spraying (IRS) [8] which has reduced the number of endophilic species [9]. Such behavioural adaptations to these current protection methods, emphasize the need for another line of defence against disease transmission. Mosquito repellents have a unique role under these conditions. Easily accessible, safe and effective mosquito repellents provide a valuable supplement to IRS and ITN use, and in areas with day-biting, exophagic vectors, this may be the only option for reducing the level of disease transmission [10].

Plant-based mosquito repellents are a viable source of material for use in protection against mosquitoes and mosquito-transmitted diseases [11] and have some advantages over the current gold-standard synthetic repellent, N,N-diethyl-m-toluamide (DEET) [10]. A variety of plants have been identified for their mosquito repellent properties through both grassroots and scientific investigations [10,11]. Volatiles from essential oils of Lamiaceae (culinary herbs), Poaceae (aromatic grasses) and Pinaceae (pine and cedar trees), are effective against various haematophagous arthropods and some essential oils, or their components, form the basis of commercial repellent formulations [11,12]. The most notable of these is *p*-menthane-3,8-diol (PMD), a hydro-distilled compound from the lemon eucalyptus plant, *Corymbia citriodora *[12].

The burning and/or hanging of fresh and dried leaves from Lamiaceae, Poaceae and Pinaceae around and within the home to provide protection against mosquito bites is widely used throughout rural Ethiopia [13,14] as well as other tropical regions [15-19]. Smoke from some of these plants is effective in repelling anopheline mosquitoes: e.g. *Ostostegia integrifolia *(90.1%) [20], *Olea europaea *(79.8%) [20], *Co. citriodora *(78.7%) [16,21] and *Ocimum suave *(44.5%) [16]. The leaves of *Oc. canum *provided 63.6% protection from mosquito bites when hung fresh in the homes in Guinea Bissau, West Africa [17]. In western Kenya, Seyoum *et al *[15,16,19] found live potted plants of *Oc. americanum, Oc. kilimandscharicum *and *Oc. suave *to be repellent providing on average of 39.7%, 44.45% and 44.45% [15,16,19] protection from bites, respectively.

In light of these studies, this investigation was carried out to evaluate the potential of volatiles from the leaves of *Co. citriodora*, *Oc. suave*, *Oc. lamiifolium*, *Os. integrifolia *and *Ol. europaea *to repel the day-biting vectors *An. arabiensis *and *Ae. aegypti*, important vectors of malaria and dengue/yellow fever in Ethiopia, respectively.

## Methods

### Experimental insects

A colony of *Ae. aegypti *[Rockefeller strain] was maintained at the Swedish University of Agricultural Sciences (SLU), Sweden. Larvae (200-300) of *Ae. aegypti *were reared in trays 15 cm wide × 30 cm long with 2-3 cm water in depth, and fed once a day on a diet of flakes (0.2-0.5 g/tray) from fish food Best Friend^® ^(Best Friend Group, Finland). In Ethiopia, *Ae. aegypti *(colony from Aklilu Lemma Institute of Pathobiology) and *An. arabiensis *(colony from WHO Malaria Control Centre) were maintained at the WHO Malaria Control Centre in Nazareth, Ethiopia; larvae were reared in trays 15 cm wide × 30 cm long in water 2-3 cm deep, and fed once a day on fed on Faffa^® ^powder (0.2-0.5 g/tray; Faffa Foods, Ethiopia). All colonies were reared under standard insectary conditions of 27 ± 2°C, 75 ± 5% R.H., L:D 12:12 h. Adults of both species were maintained in cages constructed from plastic buckets with mesh lids (20 cm diameter × 30 cm height) and were given *ad libitum *access to 10% sucrose solution. Adult non-blood-fed female mosquitoes used for experimentation were between 4 and 6 days post-emergence and starved for 12 h prior to testing.

### Odour collection

In this study, leaves of mature *Co. citriodora*, *Oc. suave *and *Oc. lamiifolium *were collected from Wondo Genet Essential Oils Research Centre in South-Central Ethiopia (latitude 7.0862, longitude 38.6190) and grown in tepid humid highland conditions (agro-ecology H3) where the major soil types are luvisols (sandy loam with PH of 7.2). Wondo Genet Essential Oils Research Centre is at an altitude of 1780 m above sea level with a temperature between 10°C and 30°C and a maximum rainfall of 2000 mm and a minimum of 700 mm. Leaves from the two other species, *Os. integrifolia *and *Ol. europaea*, were collected from Addis Zemen, Ethiopia (latitude 12.143, longitude 37.779) grown at 1975 m above sea level under the same conditions as Wondo Genet Essential Oils Research Centre stated above. Leaves from these species were chosen to be used in the volatile collections, as previous studies have demonstrated their potential as repellents [15,16,19-21]. Volatiles were collected from fresh, dried and smoking dried leaves using standard headspace sampling methods [22]. Volatiles were collected from leaves that were freshly cut and those that were dried, as well as from the smoke of burning dried leaves, representing the different ways these leaves are currently used in homes as protection against mosquitoes.

**Figure 1:**
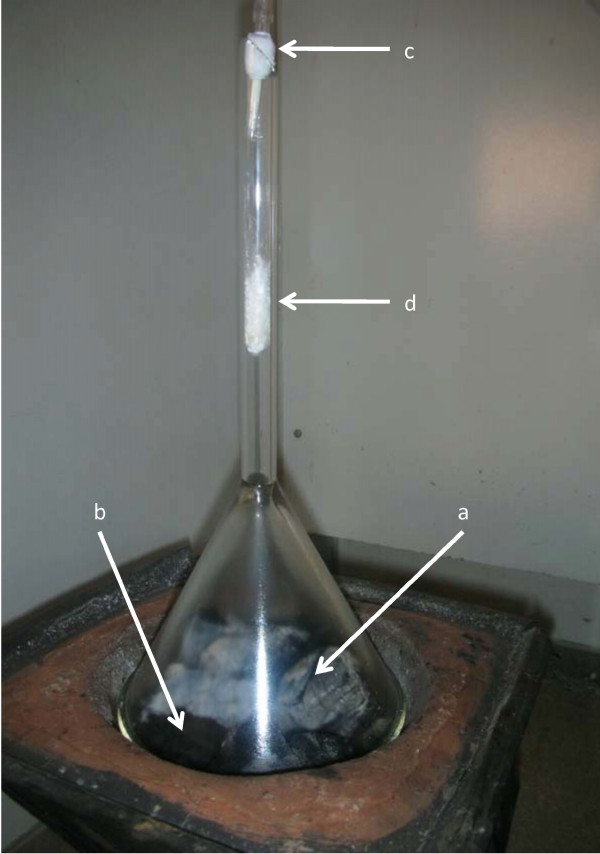
**Apparatus for collecting volatiles from the smoke of burning leaves (**a**) in a charcoal brazier (**b**)**. Volatiles were collected on a Teflon column (55 mm × 3 mm inner diameter) filled with SuperQ^® ^absorbent (35 mg, mesh 80/100) (**c**) which is protected from non-volatile smoke particulates by a glass wool plug (50 mm) (**d**) in the neck of the funnel.

Fresh and dried leaf headspace volatiles were collected for 3 h from the leaves (10 g) placed in closed glass bottles with an activated charcoal filtered air inlet and an outlet leading to the Teflon column (55 mm × 3 mm inner diameter) filled with SuperQ^® ^absorbent (35 mg, mesh 80/100, Alltech, Deerfield, IL, USA). Volatiles from the burning dried leaves (10 g) were collected by placing dried leaves onto the burning charcoal under an inverted glass funnel for 10 min allowing all leaf material to be consumed (Figure 1). In order to filter out the non-volatile particulates emitted in the smoke, a roll of glass wool (5 cm) was placed inside the tube of the funnel upstream of the volatile-collecting column filled with SuperQ^® ^absorbent as above (Figure 1). Volatile headspace was also collected from charcoal smoke alone as a bioassay control for the burned leaves, as above. After the odour collection, the volatiles were eluted by adding 300 μl hexane to the columns to obtain an extract of the volatiles. The samples were sealed in 1.5 ml glass vials (Skandinaviska GenTec AB, Sweden) and stored at-18°C until used in the behavioural studies.

### Landing bioassays with humanised membranes

**Figure 2:**
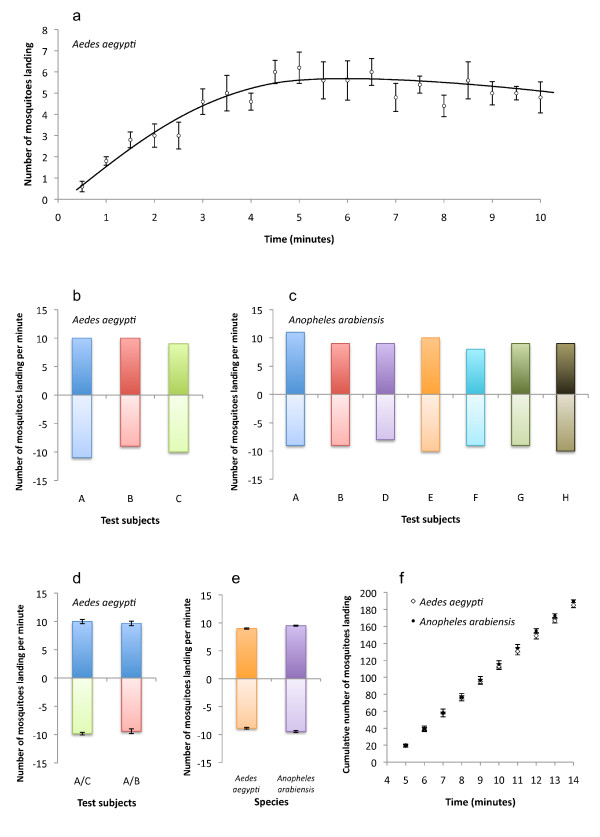
**Control experiments for the two choice landing assay with the humanised membrane**. (**a**) Rate of attraction of twenty female mosquitoes (*Aedes aegypti*) becomes constant after 4 min of exposure to untreated humanised membranes. The number of *Ae. aegypti *(**b**) and *Anopheles arabiensis *(**c**) females landing in the two-choice assay on membranes both humanised by different test subjects varies minimally. The number of *Ae. aegypti *females landing in the two-choice assay on membranes, one humanised by test subject A and the other by either test subject B or C, does not significantly differ between treatments (**d**). The number of *Ae. aegypti *and *An. arabiensis *females landing in the two-choice assay on membranes both humanised by test subject A (**e**). The rate of landing on membranes humanised by test subject A remains constant from 4 min to 14 min after both species of mosquitoes have been exposed to the membranes (**f**).

Membrane feeders, commonly used to provide a blood meal to mosquitoes in insectaries [23], were used to measure the repellence of plant volatile extracts to mosquitoes [24]. Human odour was used as an attractant and added to both control and treatment membranes (to 'humanise' them) to ensure that mosquitoes would be attracted to the membrane in the absence of any repellent compound (Figure 2) [25]. In Sweden, the behavioural response of *Ae. aegypti *was tested by using the Hemotek ("store-bought") feeding membranes (Discovery Workshops, Accrington, UK), which were rubbed for 1 min on the experimenter's palms, washed by non-perfumed soap (Lactacyd, GlaxoSmithKline, UK) 24 h before the experiment, shifting between the hands every half minute. Prior control experiments, both no choice (Figure 2b-c) and choice assays (Figure 2d), indicated that there was minimal variation in mosquito attraction to the eight people assayed. The person chosen to humanise the membranes in the experiments reported here was test subject A in order to keep the background level of attraction to the membranes consistent. Each treatment was applied at a rate of 10 μl extract per 19.625 cm^2 ^area, which Waka *et al*. [24] identified as the optimal dose per unit area. Odours from leaf headspace were diluted to 5% by using hexane giving 0.3 min equivalents (i.e. 20 s equivalents) applied for fresh and dried leaves, and 0.017 min equivalents (i.e. 1 s equivalents) for the smoke. Every treatment was tested in a two-choice landing assay in the presence of a negative control (a humanised membrane to which 10 μl of the same solvent is added). For the positive control experiments, DEET (5% and 10%) was tested in the same two-choice bioassay, also in the presence of the negative control. The solvent used in the DEET experiments was dichloromethane, an effective solvent for the long-term storage of DEET. Due to its low boiling point (40°C), dichloromethane evaporates within seconds of application to the membrane, and thus, like hexane, will not interfere with the landing bioassay results as more than 4 min have passed after application to the membrane before data collection begins. Two doses of DEET were used since they are common in commercial preparations used on both children and adults.

**Figure 3:**
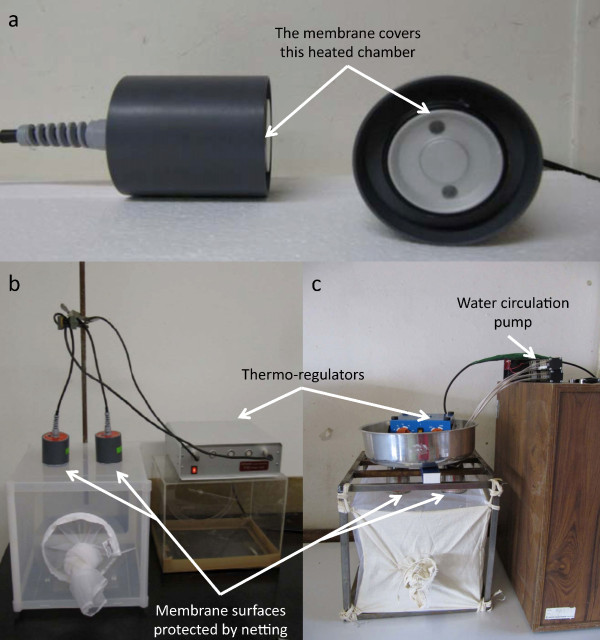
**Behavioural bioassay equipment used in this study**. Membrane feeding chambers (**a**) from the store-bought apparatus (Hemotek) are displayed here. Landing bioassay chambers from a store-bought (Hemotek) used for *Aedes aegypti *in Sweden (**b**) and a lab-constructed membrane feeding apparatus used for both *Ae. aegypti *and *Anopheles arabiensis *in Ethiopia (**c**).

The landing bioassay, carried out in Sweden, used two Hemotek chambers (6 cm diameter; Figure 3a) with humanised membranes, one with solvent added as a control and the other with the test extract, which were placed against the top netting of the cage (Figure 3b). Twenty non-blood-fed female mosquitoes were released into a 30 cm cubic gauze cage for 3 h to acclimatize and then placed in a 30 cm cubic test cage. Experiments were carried out under standard insectary and laboratory testing conditions [11] during the mosquitoes' most active periods: at dawn and dusk (06:00-08:00 and 17:00-19:00) for *Ae. aegypti *and dusk for *An. arabiensis *(17:00-19:00). After 4 min of exposure to the treatments, the time it takes to reach a constant rate of mosquito attraction to the humanised membranes (Figure 2a), the numbers of mosquitoes landing on both the extract treated and the solvent treated humanised membranes were counted at 1-min intervals for 6 min. Choice indices (CI_T _and CI_C_), as well as a repellence index (R), were determined for each treatment as follows: CI_T _= T/(T + C); CI_C _= -C/(T + C); and %R = (C-T)/C × 100% [26-28]; where T is the total number of mosquitoes landing on the extract treated humanised membrane each minute for 6 min and C is the total number of mosquitoes landing on the solvent treated humanised membrane each minute for 6 min. The experiments were replicated 3-5 times. The treatment and control chamber locations were alternated between each test to control for any potential position effect.

A bioassay chamber, similar in construction to the store-bought Hemotek apparatus (Figure 3b), was built to conduct the landing assays in Ethiopia using a metal water bath, regulatory heater, pumps and Teflon tubes (Figure 3c). The water bath was fitted with two chambers (6 cm in diameter) protruding downwards from its base and made from metal pipes (10 cm in length). Inside the bath, the heater was adjusted to 37 ± 3°C, to simulate human body temperature. In order to maintain the temperature at a uniform level throughout the bath and two chambers, the two pumps inside were connected with Teflon tubes to circulate heated water. This lab-constructed chamber also made use of the Hemotek brand membrane and both *Ae. aegypti *and *An. arabiensis *were evaluated for the repellence potential of volatiles following similar procedures as for the Hemotek landing bioassay described above.

### Chemical analysis

Volatile extracts from the leaves of all five plants were assessed using gas chromatography (GC) and, subsequently, fresh leaf extracts were evaluated by combined GC and mass spectrometry (GC-MS). Extracts were injected onto a HP 6890 gas chromatograph (Agilent Technologies, Palo Alto, CA, USA) fitted with a split-less injector (220°C) and flame ionization detector (FID) (220°C). Volatiles were separated on a fused silica capillary column (30 m × 0.25 mm inner diameter) coated with DB-WAX (df = 0.25 μm). Hydrogen was used as the mobile phase (speed 45 cm s^-1^). The oven temperature was held at 40°C for 2 min and then increased at 10°C min.^-1 ^to a final temperature of 230°C, which was held for 10 min.

The identification of active compounds in the extracts was performed by GC-MS. Each extract (2 μl) was injected onto a 6890 N gas chromatograph (Agilent Technologies) coupled to a 5975 mass spectrometer (Agilent Technologies). Compounds were separated on a similar capillary column as in the GC-analysis above. The mobile phase was helium (speed 35 cm s^-1^). The oven temperature was held at 40°C for 2 min and then increased at 10°C min^-1 ^to a final temperature of 230°C, which was held for 10 min. The identity of active compounds was determined by comparison with references from mass spectral libraries (e.g. NIST05, Agilent Technologies) and Kovats indices.

### Physiological analysis

The GC was fitted with a split at the end of the column, delivering half the effluent to the FID and the other half through a heated transfer line (230°C) into the air stream passing over the mosquito antenna mounted for electroantenno-detection (GC-EAD). A glass capillary reference electrode filled with Beadle-Ephrussi Ringer and grounded through a silver wire that was inserted into the base of the head of a mosquito. A similar recording electrode, connected to a high impedance DC amplifier with automatic baseline drift compensation, was placed over the distal cut end of an antenna. The antennal signal was stored and analysed on a PC equipped with an IDAC-card and the program EAD version 2.3 (Syntech, Kirchzarten, Germany). A repeatable response, indicating an active compound, was defined as a depolarization of the antennal signal at the same retention time in at least three trials.

Following the putative identification of antennal active compounds in fresh leaf extracts using GC-EAD and GC-MS, one compound shared by those extracts was found to be behaviourally repellent, ß-ocimene, and was used for further analysis. Various amounts of synthetic ß-ocimene were used in the electroantennographic assay to confirm its physiological activity and to determine whether it induced a dose response. ß-Ocimene was serially diluted in redistilled hexane in decadic steps (0.001-10%). Ten micro-litres of each dose was added to a 0.5 cm^2 ^piece of filter paper then placed into the end of a glass Pasteur pipette and allowed to equilibrate for at least 20 min prior use. The tip of these stimulus cartridges was then placed into the airflow over the antenna and the air diverted through the cartridge for 0.5 s. Each stimulus response has the average of two solvent blank responses, one prior and one following the stimulus pulse, subtracted to determine the antennal response to the test volatile. Antennal responses are presented as a ratio of maximal response.

### Synthetic chemicals

Synthetic volatiles are commonly used to confirm physiological and behavioural activity of compounds identified from natural extracts [22]. In these experiments, synthetic ß-ocimene was used to confirm the activity of this compound putatively identified from the odour extracts of fresh leaves. N, N-diethyl-*m*-toluamide was used as a control in the landing assays to indicate the maximal repellent behaviour of the mosquitoes. ß-ocimene (3,7-dimethyl-1,3,6-octatriene) and N,N-diethyl-*m*-toluamide (DEET), were purchased from International Flavors and Fragrances, R&D (No. 00151353; > 90%) and Sigma-Aldrich (Laborchemikalien GmbH, Seelze, Germany) respectively. Dilutions of ß-ocimene and DEET for bioassays were made in re-distilled hexane.

### Statistical analysis

The effectiveness of volatile collections (treatment) was evaluated against solvent alone (control). The repellence index (R) was estimated as %R = (C-T)/C × 100%, where C and T are the mean number of mosquitoes landing on the control and the treatment membranes, respectively [26-28]. Comparisons of repellence indices among mosquito species, landing assay type, plant species and leaf treatments were analysed by the unbalanced general linear model of the analysis of variance (ANOVA) stated as follows: A B C C*A C*B D D*A D*B D*C D*A*C D*B*C; where A is the mosquito species (*Ae. aegypti*, *An. arabiensis*), B is the bioassay (store-bought, lab-constructed), C is the plant species (*Co. citriodora*, *Oc. suave*, *Oc. lamiifolium*, *Os. integrifolia*, *Ol. europaea *and handrub control) and D is the treatment (smoke, fresh, dried and charcoal control). Dunnett's simultaneous *post hoc *tests were conducted to compare responses to the extracts with a solvent control in the landing assays, and Tukey's *post hoc *tests were used to compare among all of the responses to various doses of ß-ocimene in the physiological and behavioural assays as required using MINITAB^® ^statistical program version 14.12.0 (Minitab 2004).

## Results

### Behavioural response to the humanised membrane

Under no-choice conditions with a single humanised membrane, the rate of attraction of *Ae. aegypti *females stabilised after 4 min (5 ± 1 per minute; Figure 2a). Mosquitoes were tested for attraction to membranes humanised by eight different test subjects. In a no-choice assay with two humanised membranes, attraction to membranes humanised by different test subjects was not significantly different in either species (*Ae. aegypti *χ^2 ^0.1360, *df *2; *An. arabiensis *χ^2 ^0.3625, *df *6; Figure 2b and 2c). In a two-choice assay, test subject A was not significantly more attractive than either test subject B (paired *t*-test *t *= 0.3492, *df *5) or C (paired *t*-test *t *= 0.5058, *df *11) to the *Ae. aegypti *females (Figure 2d). Test subject A was therefore chosen to humanise all membranes in the subsequent experiments. In no-choice assays with two membranes humanised by test subject A, the landing rate per minute over 10 min did not differ between the species (unpaired *t*-test *t *= 1.247, *df *18; Figure 2e). The rate of attraction to the humanised membranes was determined to be constant in *Ae. aegypti *(R^2 ^= 0.99987) and *An. arabiensis *(R^2 ^= 0.99981) over 10 min (Figure 2f) following the 4-min acclimatisation period (Figure 2a). Neither *Ae. aegypti *nor *An. arabiensis *differed in the number of landings made on humanised membranes with either the extract from the charcoal smoke headspace collection or solvent control (data not shown).

### Behavioural response to plant extracts

**Figure 4:**
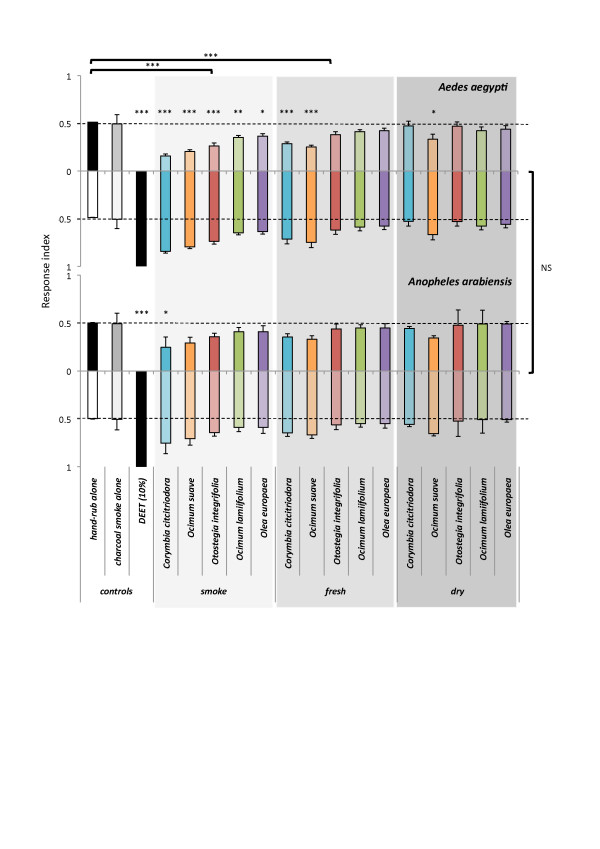
**Leaf volatiles repel mosquitoes**. Female *Aedes aegypti *and *Anopheles arabiensis *(N = 9) landing behaviour was tested in response to volatile extracts from five plants traditionally used to repel mosquitoes. Volatile extracts have been taken from burning, fresh, and dried leaves. N,N-Diethyl-meta-toluamide (DEET, 10%) was used as a control to indicate maximal repellent response. Extracts were added to a hand-rubbed, heated membrane and compared to a hand-rubbed, heated membrane alone in a two-choice assay. Significance is determined by two-way analysis of variance (ANOVA) with Dunnett's simultaneous *post hoc *test at *P *< 0.05 unless otherwise indicated (** *P *< 0.01; *** *P *< 0.001). Error bars represent the standard error of the mean (SEM).

The two-choice landing bioassays using membrane feeders (Figure 3) were conducted under laboratory conditions on *Ae. aegypti *and *An. arabiensis *females. Of the mosquitoes that were activated to fly (≥ 80%), the number of mosquitoes landing on either of the proffered membranes each minute for 6 min allowed for the calculation of choice indices associated with the control (CI_C_) and treated (CI_T_) membranes (Figure 4) as well as the repellence index (R).

A general linear model (GLM) for the analysis of variance (ANOVA) of the repellence indices was developed including four factors (bioassay, mosquito species, plant species and leaf treatment). Prior to the generation of the GLM, the repellence indices were determined to follow a normal distribution (D'Agostino-Pearson normality test, *P *> 0.05). This model determined that the landing behaviour of *Ae. aegypti *did not differ between the store-bought and lab-constructed bioassays (F = 0.06; DFn = 1; DFd = 144; *P *= 0.81; Figure 4). The pattern of landing behaviour of the female mosquitoes, whether *Ae. aegypti *or *An. arabiensis*, in response to all the extracts in the two-choice assay did not significantly differ (F = 2.98; DFn = 1; *P *= 0.087; Figure 4). The GLM indicated that there was no significant interaction among any of the factors (mosquito*plant, F = 0.15, DFn = 5, *P *= 0.98; bioassay*treatment, F = 0.61, DFn = 2, *P *= 0.55; plant*bioassay, F = 0.05, DFn = 5, *P *= 1.00; bioassay*treatment, F = 0.95, DFn = 15, *P *= 0.50; mosquito*plant*treatment, F = 0.17, DFn = 15, *P *= 1.00; bioassay*plant*treatment, F = 0.12, DFn = 15, *P *= 1.00). Therefore, further comparative analyses of female mosquito responses were made considering plant species (F = 5.93, DFn = 5, *P *< 0.001) and manner of leaf treatment (F = 14.90, DFn = 2, *P *< 0.001) alone.

In the two-choice assay, the landing of female *Ae. aegypti *on the control humanised membranes was significantly more than on the humanised membranes treated with the smoke extracts of all five plant species (*t*-values 5.96, 5.45, 4.63, 3.30 and 3.00; *P*-values < 0.001, < 0.001, < 0.001, 0.011 and 0.025), the fresh extracts of *Co. citriodora *(*t*-value 4.16, *P *< 0.001) and *Oc. suave *(*t*-value 4.63, *P *< 0.001), and the extract of dry leaves from *Oc. suave *(*t*-value 2.83, *P *= 0.038; Figure 4). While in *An. arabiensis *this overall trend in behaviour appears similar to *Ae. aegypti *(Figure 4), only the smoke extract from *Co. citriodora *results in a significant decrease in landing compared to the control (*t*-value 2.56, *P *= 0.050). The standard repellent used for this two-choice bioassay in the background of human odour was DEET at two concentrations (5 and 10%), which resulted in 100 ± 0% mosquito repellence in the landing bioassay. All extracts appeared less potent repellents than DEET (*P *> 0.001; Figure 4).

### Gas chromatography and mass spectroscopy

**Figure 5:**
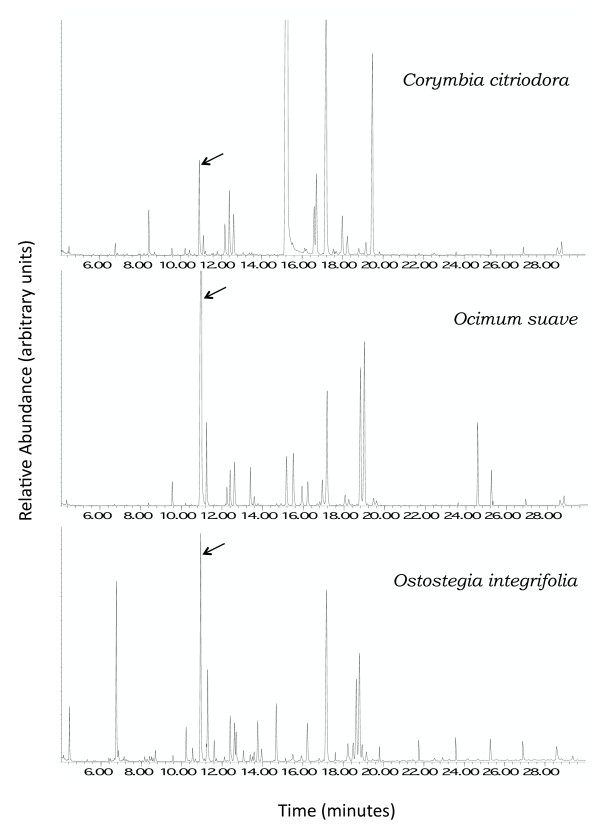
**Identification of ß-ocimene**. Total ion chromatograms (TIC) of the headspace collected from fresh leaves of plants whose extracts were shown to be repellent in two-choice assays. The arrows indicate the peak that has since been identified as Z-ß-ocimene using mass spectrometry.

The smoked leaf extracts resulted in highly complex chromatograms that were difficult to interpret, while dried leaf extracts appeared to have very low levels of volatile compounds on the chromatogram. For these reasons, the investigations focused on the fresh leaf extracts (Figure 5). The GC-EAD was performed using the fresh leaf extract on *Ae. aegypti *antennae (data not shown). The repeated physiological response of the antennae to one GC peak (10.94 min.) was shared amongst the most repellent headspace extracts, *Co. citriodora*, *Oc. suave *and *Os. integrifolia*, and thus prompted further investigation. The putative identification of this compound through GC-MS determined it was Z-ß-ocimene. The relative amounts of Z-ß-ocimene present in the headspace of these three species, and not found in the other two, reflected the headspace repellence determined in the bioassays (Figures 4 and 5). Although other GC peaks were shared among these three species (e.g. at 17.5 min.), none of these showed consistent, repeatable physiological responses during GC-EAD analyses, therefore these peaks were not pursued further in this study.

### Synthetic ß-ocimene

**Figure 6:**
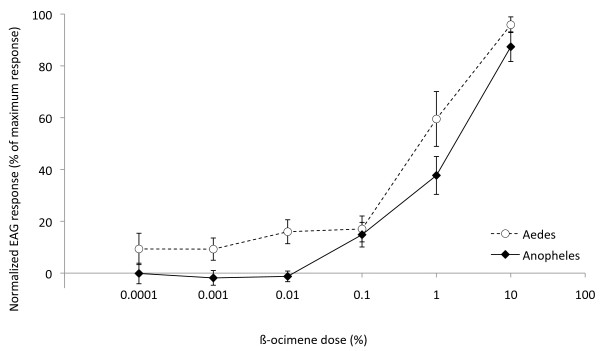
**Antennal response to synthetic ß-ocimene**. Female *Anopheles arabiensis *(diamonds; n = 6) and B. *Aedes aegypti *(circles; n = 4) antennal response to ß-ocimene using an electroantennogram (EAG).

A mixture of both geometric isomers (E- and Z-) of ß-ocimene is detected by the antennae of females of both mosquito species under investigation, *An. arabiensis *and *Ae. aegypti*. Using electroantennogram (EAG) recordings, the antennal response of *An. arabiensis *and *Ae. aegypti *to synthetic ocimene was found to be dose dependent (r^2 ^= 0.9325 and r^2 ^= 0.998, respectively; F = 26.8, DFn = 6, *P *< 0.001; Figure 6) with a threshold response for *An. arabiensis *between 0.01 and 0.1% and for *Ae. aegypti *between 0.1 and 1%. The estimated ED_50 _for physiological response was approximately 0.65% and 1% for *Ae. aegypti *and *An. arabiensis*, respectively, and this difference is reflected in the marginally significant increase in sensitivity of antennal response in *Ae. aegypti *compared with *An. arabiensis *at 1% ß-ocimene (Tukey *post hoc P *= 0.050; Figure 6).

**Figure 7:**
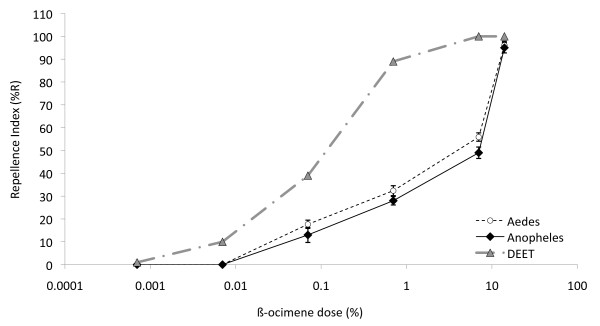
**Behavioural response to synthetic ß-ocimene**. Female *Aedes aegypti *(circles, n = 10) and *Anopheles arabiensis *(diamonds, n = 5) landing behaviour in response to ß-ocimene (circles and diamonds) and DEET (triangles, n = 3) added to a hand-rubbed, heated membrane compared to a hand-rubbed, heated membrane alone in a two-choice assay.

In the landing assay, more *An. arabiensis *and *Ae. aegypti *females land on solvent-treated rather than synthetic ß-ocimene treated, human-scented membranes in a dose dependent manner (Figure 7; r^2 ^= 0.945, F = 12.27, DFn = 6, *P *< 0.001 and r^2 ^= 0.926, F = 582.0, *P *< 0.001, respectively). Both *Ae. aegypti *and *An. arabiensis *were strongly repelled by 14% ß-ocimene (96 ± 5.3% and 95 ± 5.1% respectively), which was not significantly different from the response of *Ae. aegypti *to 10% DEET (ANOVA; F = 2.586; DFn = 6, *P *> 0.05). The behavioural sensitivity of both mosquito species to DEET is approximately 10 times that of ß-ocimene, as estimated by ED_50 _(0.1% DEET and 1-2% ß-ocimene). While there is no significant interaction between species and dose, the two mosquito species appear to behave significantly differently to ß-ocimene (2-way ANOVA; F = 7.279; DFn = 6, *P *= 0.010) with *Ae. aegypti *being marginally more sensitive to 0.7% ß-ocimene (Tukey *post hoc P *= 0.05; Figure 7).

## Discussion

The present study demonstrates that odours from the leaves of five plants traditionally used in Ethiopia as protection from mosquito bites have repellent properties. The headspace extracts of smoke from burning these leaves were more repellent than those from fresh or dried leaves of the same plant species to host-seeking (i.e. non-blood-fed) female mosquitoes of both *An. arabiensis *and *Ae. aegypti*, the primary malaria and dengue/yellow fever vectors in Ethiopia, respectively. Previous studies, in which *Os. integrifolia *[20], *Ol. europaea *[20], *Co. citriodora *[16,21] and *Oc. suave *[16] were burned to repel mosquitoes, have also demonstrated a large reduction in the number of mosquitoes landing. Not only these, but there are also many other examples of burning leaves to decrease the number of mosquitoes in the house, some of which have also resulted in the reduction of other arthropod vector densities indoors, such as the sand fly and black fly [29,30]. According to Hoek *et al *[31] there is a significantly lower risk of malaria in households using traditional smokes and fumigants, such as the burning leaves in this study, compared with those that did not. These previous studies on the effects of plant-derived smoke on arthropod vectors support our present findings, which clearly demonstrate the potential for the use of combustion-released volatiles against mosquitoes.

Some possible mechanisms for the action of plant-derived smoke have been proposed [1]. The smoke may disguise human kairomone cues used by the vectors to target their hosts, it may disrupt the convection currents essential for mosquito host location, and/or the burning of these leaves may release volatile compounds that act as repellents or irritants against the mosquito. Smoke from common firewood has been reported to be a mosquito repellent by deterring mosquitoes from roosting in houses [30,32] suggesting that masking and current disruption play a role in the efficacy of leaf smoke/fumigants. The current study, however, investigated the third premise and has demonstrated that the protection against the mosquitoes resulting from the burning of dried leaves appears to be due to the release of volatile compounds during combustion. Moreover, the background odours from the charcoal fuel used for smoke odour collection demonstrated no repellent properties when tested in the landing bioassay. Thus, at least a portion of the repellent potential of smoke headspace originates from the plant volatiles themselves. Heat, convection currents and particulates in the smoke may also play a role in the protection provided by plant-derived smoke as discussed above, but this was not tested in this study.

Mosquito membrane feeders have previously been used to test arthropod repellence in bioassays using animal blood or skin as the background attractant for the mosquitoes [24,33]. In this study, an alternative method for screening repellents was found to be 'humanising the membranes' by rubbing the feeding membrane with human hands to transfer the appropriate host odours. One advantage with the humanised membranes is that the results obtained from these bioassays relate directly to the efficacy of repellent for human rather than animal protection. Emanations found associated with human hands, such as sweat, are detected by [34,35], and shown to be behaviourally attractive to, mosquitoes [36]. Also, humanising the membranes is fast, ethically safe and can be easily done under laboratory or field conditions. The two landing bioassay arenas used in this study, one store bought (Hemotek membrane feeder, Discovery Workshops, UK) and the other self assembled (F.F. Dube, Addis Ababa University, ET), were found to be equally efficient, demonstrating again the simplicity and ease of use of this technique.

Among the plant species tested, the headspace extracts from burning the dried leaves of *Co. citriodora *and *Oc. suave *were the strongest host-seeking female mosquito repellent (> 65%) both for *An. arabiensis *and *Ae. aegypti *in the laboratory. The headspace extracts of *Co. citriodora *and *Oc. suave *fresh leaves were also shown to be significantly repellent (> 50%) while the dried leaves of none of the species were strongly repellent (< 30%). The smoke from leaves of *Co. citriodora *was previously suggested as a strong mosquito repellent in controlled semi-field studies using volatiles expelled through heating the leaves on metal plate [15,16,21]. The increased repellent potency of the headspace of burning leaves may be due to the increased release rate of repellent volatile compounds, either already present in the fresh/dried leaf and/or created during the combustion process.

That repellent compounds are present in the leaves of many plant species has been well documented [12,37,38]. Both the juices and the essential oil extracts from leaves have arthropod repellent properties. For example, the essential oil extracted from *Co. citriodora *can act directly as a natural insect repellent to provide protection against mosquitoes and other harmful arthropods [37]. In fact, PMD, a compound found in the essential oil of *Co. citriodora *[12], is the only naturally derived active ingredient to be certified as an insect repellent in Europe and the USA that is commercially available. The juice from the leaves of *Oc. suave *and *Oc. canum *spread on the legs of human volunteers has approximately 50% reduction of mosquitoes landing [38].

Z-ß-ocimene was identified from the fresh leaf headspace extracts of *Co. citriodora*, *Oc. suave *and *Os. integrifolia *as an effective mosquito repellent compound in the humanised membrane laboratory repellence assay. A mixture of both geometric isomers of ß-ocimene (14%) was not significantly different in repellent activity from DEET (10%) over 6 min in the landing assay. ß-ocimene has been identified previously in volatile emissions of *Co. citriodora *(ca. 10% ß-ocimene) [39]. In the search for repellents, but more often for insecticides, ß-ocimene has been identified as a major component of leaf essential oils, e.g. *Oc. suave *(13.5% Z-ß-ocimene, [40]) as well as other plant species [41-44]. While common practice assesses the entire essential oil for its bioactivity, ß-ocimene itself has been shown to be an effective insecticide against some crop pests [45] and honeybee mites [46]. Essential oils containing a high proportion of ß-ocimene have been assessed for larvicidal activity against mosquitoes [43,44,47-49], however until now, ß-ocimene alone has not been evaluated for its efficacy as a repellent of host-seeking mosquitoes. The results indicate that ß-ocimene is an interesting biologically active volatile that should be included in further examinations of plant-derived mosquito repellents, including confirmation of mosquito-repellent activity using conventional repellence assays [10,11] as well as duration of action and efficacy in blends with other naturally derived compounds. As ß-ocimene is a highly volatile compound, its formulation for use as a skin-applied repellent is critical when it comes to its duration of action. Some formulation methods to reduce the volatility of such compounds have been described [50]. For example, the addition of a large molecule such as vanillin can substantially extend the duration of action of other natural, but highly volatile, repellents [50]. The common use of ß-ocimene as a pleasing scent in commercial products, e.g. alcohol- and cream-based perfumes, which need to continue to release their scent over a number of hours after application, bodes well for the possibility of finding long-lasting, not to mention pleasant smelling, repellent formulations containing ß-ocimene. ß-ocimene may be a cheap and locally available mosquito repellent with an inoffensive odour that government and regulatory bodies, such as the EU EFSA [51] and US FDA [52], have already certified as safe for use in products applied to human skin at concentrations up to 20% (with the recommended dose of 5% to prevent the possibility of skin irritation or sensitisation [51]). These concentrations, currently used in many commercially available products (e.g. perfume, soap and deodorant) appear to be within the repellent activity against both mosquito species tested in the laboratory.

## Conclusions

Therefore, further studies are proposed to characterize the repellent potential of ß-ocimene and to identify other potential volatiles from headspace leaf extracts of *Co. citriodora *and *Oc. suave*. The use of a two-choice landing bioassay with humanised membranes providing the background emanation promises to be an efficient screening technique for the assessment of ecologically relevant potential mosquito repellents, be they headspace extracts, essential oils or synthetic compounds. While this technique represents a potentially useful repellent screening technique that does not require the exposure of humans to the vectors, further tests in parallel with conventional techniques are advised.

## Competing interests

The authors declare that they have no competing interests.

## Authors' contributions

FFD carried out the behavioural, GC, GC-EAD and GC-MS experiments, and constructed the membrane-landing assay in Addis Ababa. GB carried out the GC and GC-MS analyses together with FFD. KT carried out the dose response EAG experiments with ß-ocimene. ES, together with RI and SRH conceived the study and participated in the study design. ES and HT coordinated the Ethiopian studies, while RI and SRH did the same in Sweden. SRH performed the statistical analyses. FFD and SRH drafted the manuscript. All authors read and approved the final manuscript.

## References

[B1] Moore SJ Lenglet AD Wilcox M, Bodeker G, Rasoanaivo P An overview of plants used for insect repellents Traditional Medicinal Plants and Malaria 2004 London: CRC Press, Taylor and Francis 344 363

[B2] Chen LH Wilson ME Schlagenhauf P Prevention of Malaria in long-term travellers J Am Med Assoc 2006 296 2234 2244 10.1001/jama.296.18.2234 17090770

[B3] Choi HW Breman JG Teutsch SM Liu S Hightower AW Sexton JD The effectiveness of insecticide-impregnated bednets in reducing cases of malaria infection: a meta-analysis of published results Am J Trop Med Hyg 1995 52 377 382 7771600 10.4269/ajtmh.1995.52.377

[B4] Gonzalez JO Kroeger K Avina AI Pabon E Wash resistance of insecticide-treated materials Trans R Soc Trop Med Hyg 2002 96 370 375 10.1016/S0035-9203(02)90363-9 12497971

[B5] Munhenga G Masendu HT Brooke BD Hunt RH Koekemoer LK Pyrethroid resistance in the major malaria vector *Anopheles arabiensis *from Gwave, a malaria-endemic area in Zimbabwe Malar J 2008 7 247 10.1186/1475-2875-7-247 19038063 PMC2613911

[B6] Yohannes M Boele E Early biting rhythm in the afro-tropical vector of malaria, *Anopheles arabiensis*, and challenges for its control in Ethiopia Med Vet Entomol 2011 25 doi: 10.1111/j.1365-2915.2011.00955.x 10.1111/j.1365-2915.2011.00955.x 21410494

[B7] Beebe NW Bakotee B Ellis JT Cooper RD Differential ecology of *Anopheles punctulatus *and three members of the *Anopheles farauti *complex of mosquitoes on Guadalcanal, Solomon Islands, identified by PCR-RPLP analysis Med Vet Entomol 2000 14 308 312 10.1046/j.1365-2915.2000.00248.x 11016439

[B8] Tadei WP Thatcher BD Santos JMM Scarpassa VM Rodrigues IB Rafael MS Ecologic observations on Anopheline vectors of malaria in the Brazilian Amazon Am J Trop Med Hyg 1998 59 325 335 9715956 10.4269/ajtmh.1998.59.325

[B9] Suthas N Phorn S Udom C Cullen JR The behavior of *Anopheles minimus *Theobald (Diptera: Culicidae) subjected to differing levels of DDT selection pressure in northern Thailand Bull Entomol Res 1986 76 303 312 10.1017/S0007485300014772

[B10] Moore SJ Wilcox M, Bodeker G, Rasoanaivo P Guidelines for Studies on plant-based insect repellents Traditional Medicinal Plants and Malaria 2004 London: CRC Press, Taylor and Francis 365 372

[B11] Maia MF Moore SJ Plant-based insect repellents: a review of their efficacy, development and testing Malar J 2011 10 S11 10.1186/1475-2875-10-S1-S11 21411012 PMC3059459

[B12] Carroll SP Loye J PMD, a registered botanical mosquito repellent with deet-like efficacy J Amer Mosq Control Assoc 2006 22 507 514 10.2987/8756-971X(2006)22[507:PARBMR]2.0.CO;2 17067054

[B13] Karunamoorthi K Mulelam A Wassie F Assessment of knowledge and usage custom of traditional insect/mosquito repellent plants in Addis Zemen Town, South Gonder, North Western Ethiopia J Ethnopharmacol 2009 121 49 53 10.1016/j.jep.2008.09.027 18977426

[B14] Karunamoorthi K Ilango K Endale A Ethnobotanical survey of knowledge and usage custom of traditional insect/mosquito repellent plants among the Ethiopian Oromo ethnic group J Ethnopharmacol 2009 125 224 229 10.1016/j.jep.2009.07.008 19607902

[B15] Seyoum A Palsson K Kung'a S Kabiru EW Lwande W Killeen GF Hassanali A Knols BGJ Traditional use of mosquito-repellent plants in western Kenya and their evaluation in semi-field experimental huts against *Anopheles gambiae*: ethnobotanical studies and application by thermal expulsion and direct burning Trans R Soc Trop Med Hyg 2002 96 225 231 10.1016/S0035-9203(02)90084-2 12174767

[B16] Seyoum A Killeen GF Kabiru EW Knols BG Hassanali A Field efficacy of thermally expelled or live potted repellent plants against African malaria vectors in western Kenya Trop Med Int Health 2003 8 1005 1011 10.1046/j.1360-2276.2003.01125.x 14629767

[B17] Moore SJ Lenglet A Hill N Debboun M, Frances SP, Strickman D Plant-based insect repellents Insect Repellents: Principles, Methods and Uses 2006 Boca Raton: CRC Press, Taylor and Francis Group 275 303

[B18] Palsson K Jænson TG Plant products used as mosquito repellents in Guinea Bissau, West Africa Acta Trop 1999 72 39 52 10.1016/S0001-706X(98)00083-7 9924960

[B19] Seyoum A Kabiru EW Wande WL Killeen GF Hassanali A Knols BGJ Repellency of live potted plants against *Anopheles gambiae *from human baits in semi-field experiments huts Am J Trop Med Hyg 2002 67 191 195 12389946 10.4269/ajtmh.2002.67.191

[B20] Karunamoorthi K Mulelam A Wassie F Laboratory evaluation of traditional insect/mosquito repellent plants against *Anopheles arabiensis*, the predominant malaria vector in Ethiopia Parasitol Res 2008 103 529 534 10.1007/s00436-008-1001-9 18493796

[B21] Dugassa S Medhin G Balkew M Seyoum A Gebre Michael T Field investigation on the repellent activity of some aromatic plants by traditional means against *Anopheles arabiensis *and *An. pharoensis *(Diptera: Culicidae) around Koka, central Ethiopia Acta Trop 2009 112 38 42 10.1016/j.actatropica.2009.06.002 19539591

[B22] Bengtsson JM Wolde-Hawariat Y Khbaish H Negash M Jembere B Seyoum E Hansson BS Larsson MC Hillbur Y Field Attractants for *Pachnoda interrupta *Selected by Means of GC-EAD and Single Sensillum Screening J Chem Ecol 2009 35 1063 1076 10.1007/s10886-009-9684-7 19768509 PMC2847173

[B23] Cosgrove JB Wood RJ Probing and gorging responses of three mosquito species to a membrane feeding system at a range of temperatures J Am Mosq Cont Assoc 1995 11 339 342 8551304

[B24] Waka M Hopkins RJ Curtis C Ethnobotanical survey and testing of plants traditionally used against hematophagous insects in Eritrea J Ethnopharmacol 2004 95 95 101 10.1016/j.jep.2004.07.003 15374613

[B25] Andreasen MH Bilrtles A Curtis CF Wood RJ Enhanced blood feeding of *Anopheles *mosquitoes (Diptera: Culicidae) through membranes with applied host odour Bull Entomol Res 2004 94 291 295 15191630 10.1079/ber2004295

[B26] Sharma VP Ansari MA Personal protection from mosquitoes (Diptera: Culicidae) by burning neem oil in kerosene J Med Entomol 1994 31 3 505 507 7914543 10.1093/jmedent/31.3.505

[B27] Yap HH Jahangir K Chong ASC Adanan CR Chong NL Malik YA Rohaizat B Field efficacy of a new repellent, KBR 3023, against *Aedes albopictus *(SKUSE) and *Culex quinquefasciatus *(SAY) in a tropical environment J Vector Ecol 1998 23 1 62 68 9673931

[B28] Chio EH Yang EC A bioassay for natural insect repellents J Asia Pacific Entomol 2008 4 225 227

[B29] Moore SJ Debboun M Debboun M, Frances SP, Strickman D History of Insect Repellents Insect Repellents: Principles, Methods and Uses 2006 Boca Raton: CRC Press, Taylor and Francis Group 3 29

[B30] Biran A Smith L Lines J Ensink J Cameron M Smoke and malaria: are interventions to reduce exposure to indoor air pollution likely to increase exposure to mosquitoes? Trans R Soc Trop Med Hyg 2007 101 1065 1071 10.1016/j.trstmh.2007.07.010 17888474

[B31] Hoek W Konradsen F Dijkstra DS Amerasinghe PH Amerasinghe FP Risk factors for malaria: a micro-epidemiological study in a village in Sri Lanka Trans R Soc Trop Med Hyg 1998 92 265 269 10.1016/S0035-9203(98)91003-3 9861392

[B32] Kweka EJ Mosha FW Lowassa A Mahande AM Mahande MJ Massenga CP Tenu F Lyatuu EE Mboya MA Temu EA Longitudinal evaluation of *Ocimum *and other plants effects on the feeding behavioral response of mosquitoes (Diptera: Culicidae) in the field in Tanzania Parasite Vect 2008 42 1 8 10.1186/1756-3305-1-42 PMC2577633 18945343

[B33] Debboun M Wagman J *In vitro *repellency of N, N-diethyl-3- methylbenzamide and N,N-diethylphenylacetamide analogues against *Aedes aegypti *and *Anopheles stephensi *(Diptera: Culicidae) J Med Entomol 2004 41 430 434 10.1603/0022-2585-41.3.430 15185946

[B34] Ghaninia M Ignell R Hansson BS Functional classification and central nervous projections of olfactory receptor neurons housed in antennal trichoid sensilla of female yellow fever mosquitoes, *Aedes aegypti* European J Neurosci 2007 26 1611 1623 10.1111/j.1460-9568.2007.05786.x PMC2121139 17880395

[B35] Hill SR Hansson BS Ignell R Characterization of antennal trichoid sensilla from female southern house mosquito, *Culex quinquefasciatus *Say Chem Senses 2009 34 231 252 19153252 10.1093/chemse/bjn080

[B36] Braks MAH Takken W Incubated human sweat but not fresh sweat attracts the malaria mosquito *Anopheles gambiae sensu stricto* J Chem Ecol 1999 25 663 672 10.1023/A:1020970307748

[B37] Yang YC Choi HC Choi WS Clark JM Ahn YJ Ovicidal and adulticidal activity of *Eucalyptus globulus *leaf oil terpenoids against *Pediculus humanus capitis *(Anoplura: Pediculidae) J Agri Food Chem 2004 52 2507 2511 10.1021/jf0354803 15113148

[B38] Lukwa N Nayzema NZ Curtis CF Mwaiko GL Chandiwana SK People's perceptions about malaria transmission and control using mosquito repellent plants in a locality in Zimbabwe Cent African J Med 1999 45 64 68 10.4314/cajm.v45i3.8456 10565064

[B39] Zini CA Augusto F Christensen E Smith BP Bastos Caramão E Pawliszyn J Monitoring biogenic volatile compounds emitted by *Eucalyptus citriodora *using SPME Anal Chem 2001 73 4729 4735 10.1021/ac0103219 11605854

[B40] Chogo JB Crank G Chemical composition and biological activity of the Tanzanian plant *Ocimum suave* J Nat Prod 1981 44 308 311 10.1021/np50015a012 6114991

[B41] Jænson TGT Pålsson K Borg-Karlsona A-K Evaluation of extracts and oils of mosquito (Diptera: Culicidae) repellent plants from Sweden and Guinea-Bissau J Med Entomol 2006 43 113 119 10.1603/0022-2585(2006)043[0113:EOEAOO]2.0.CO;2 16506457

[B42] Gillij YG Gleisera RM Zygadlo JA Mosquito repellent activity of essential oils of aromatic plants growing in Argentina Bioresource Technol 2008 99 2507 2515 10.1016/j.biortech.2007.04.066 17583499

[B43] Dharmagadda VSS Naik SN Mittal PK Vasudevan P Larvicidal activity of *Tagetus patula *essential oil against three mosquito species Bioresource Technol 2005 96 1235 1240 10.1016/j.biortech.2004.10.020 15734310

[B44] Senthilkumar A Kannathasan K Venkatesalu V Chemical constituents and larvicidal property of the essential oil of *Blumea mollis *(D. Don) Merr. against *Culex quinquefasciatus* Parasitol Res 2008 103 959 962 10.1007/s00436-008-1085-2 18566831

[B45] Ogendoa JO Kostyukovsky M Ravidc U Matasyohd JC Denge AL Omoloa EO Kariukie ST Shaaya E Bioactivity of *Ocimum gratissimum *L. oil and two of its constituents against five insect pests attacking stored food products J Stored Products Res 2008 44 328 334 10.1016/j.jspr.2008.02.009

[B46] Ruffinengo S Eguaras M Floris I LD_50 _and repellent effects of essential oils from Argentinian wild plant species on *Varroa destructor* J Eco Entomol 2005 98 651 655 10.1603/0022-0493-98.3.651 16022288

[B47] Cheng SS Liu JY Tsai KH Chen WJ Chang ST Chemical composition and mosquito larvicidal activity of essential oils from leaves of different *Cinnamomum osmophloeum provenances* J Agric Food Chem 2009 52 4395 4400 10.1021/jf0497152 15237942

[B48] Conti B Canale A Bertoli A Gozzini F Pistelli L Essential oil composition and larvicidal activity of six Mediterranean aromatic plants against the mosquito *Aedes albopictus *(Diptera: Culicidae) Parasitol Res 2010 107 1455 1461 10.1007/s00436-010-2018-4 20697909

[B49] Zhu L Tian Y Chemical composition and larvicidal activity of *Blumea densiflora *essential oils against *Anopheles anthropophagus*: a malarial vector mosquito Parasitol Res 2011 DOI: 10.1007/s00436-011-2388-2 10.1007/s00436-011-2388-2 21556689

[B50] Tawatsin A Wratten SD Scott RR Thavara U Techadamrongsin Y Repellency of volatile oils from plants against three mosquito vectors J Vector Ecol 2001 26 76 82 11469188

[B51] EFSA Flavoring Group Evaluation 25, Revision 1 (FGE.25Rev1): Aliphatic and aromatic hydrocarbons from chemical group 311 EFSA Panel on Food Contact Materials, Enzymes, Flavorings and Processing Aids (CEF) EFSA Journal 2010 8 1334

[B52] FDA Part 172: Food additives permitted for direct addition to food for human consumption, Subpart F--Flavoring Agents and Related Substances Sec 172.515 Synthetic flavoring substances and adjuvants 2010

